# Supplementary material for: A Theoretical Study on the Degradation Mechanism, Kinetics, and Ecotoxicity of Metronidazole (MNZ) in •OH- and SO4•−-Assisted Advanced Oxidation Processes
Source: Toxics. 2023 Sep 20;11(9):796. doi: 10.3390/toxics11090796 (PMC10535747; doi:10.3390/toxics11090796)
Supplement: Supplementary file 1 [file toxics-11-00796-s001.zip › toxics-2576171-supplementary.pdf]

A theoretical study on the degradation mechanism, kinetics, and ecotoxicity of metronidazole (MNZ) in •OH- and $SO_4^{\bullet-}$-assisted advanced oxidation processes

Jingyu Sun[1*], Ruijun Chu[1], Zia Ul Haq Khan[2]

[1] *Hubei Key Laboratory of Pollutant Analysis & Reuse Technology, College of Chemistry and Chemical Engineering, Hubei Normal University, Cihu Road 11, Huangshi 435002, China*

[2]*Department of Chemistry, COMSATS University Islamabad, Park Road, Islamabad 45550, Pakistan*

∗ Corresponding author. Email address: sunjy@hbnu.edu.cn Tel.:/Fax: 0714-6515602

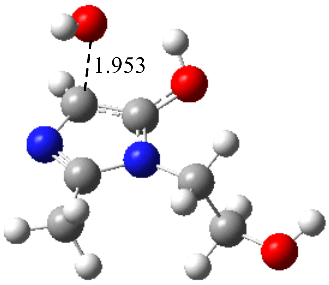

TS5

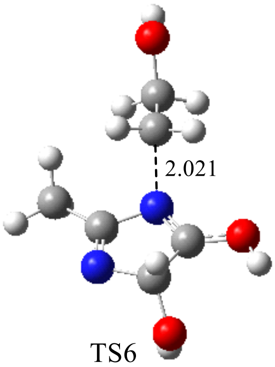

TS6

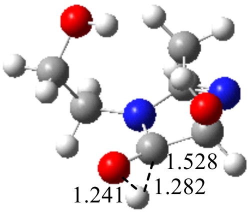

TS7

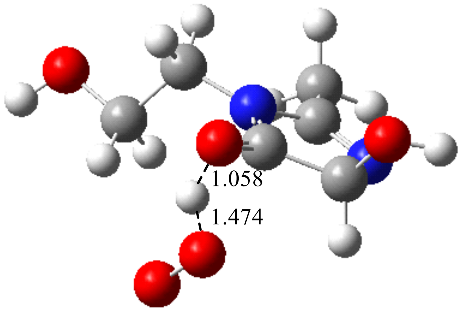

TS8

Figure S1. Optimized geometries of the transition states (TSs) involved subsequent degradation of M-P. Bond lengths are in angstroms.

Table S1. The condensed Fukui function values of M-P product.

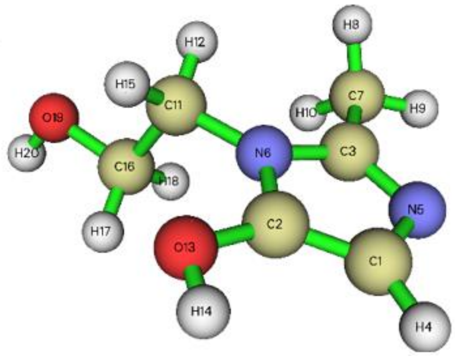

**Molecule**	**Atom**	f_k^-	f_k^+	f_k^0
	1(C)	0.1596	0.0480	**0.1038**
	2(C)	0.1223	0.0453	0.0838
	3(C)	0.1179	0.0716	0.0948
	4(H)	0.0630	0.0363	0.0497
	5(N)	0.0923	0.0788	0.0855
	6(N)	0.0318	0.0317	0.0318
	7(C)	0.0336	0.0290	0.0313
	8(H)	0.0426	0.0338	0.0382
	9(H)	0.0298	0.0305	0.0301
	10(H)	0.0396	0.0302	0.0349
	11(C)	0.0103	0.0219	0.0161
	12(H)	0.0194	0.0263	0.0228
	13(O)	0.1185	0.0708	0.0946
	14(H)	0.0427	0.1564	0.0995
	15(H)	0.0184	0.0292	0.0238
	16(C)	0.0061	0.0264	0.0162
	17(H)	0.0048	0.0173	0.0111
	18(H)	0.0062	0.0192	0.0127
	19(O)	0.0221	0.0627	0.0424
	20(H)	0.0191	0.1346	0.0768

Table S2 Gibbs free energies (Δ*G*) and relative energies (Δ*E*) for reaction intermediates (IM), products, and transition states (TS) of MNZ with HO• and $SO_4^{\bullet-}$ initial reaction at the double level of M06-2X/6-31+G(3df, 2p)//M06-2X/6-31+G(d, p).

Species	Δ*G*	Δ*E*	Species	Δ*G*	Δ*E*
RAF-1TS	5.89	-3.70	RAF-5TS	6.78	-7.16
RAF-2TS	10.34	1.90	RAF-6TS	10.13	-3.57
RAF-3TS	26.92	17.57	RAF-7TS	37.12	23.67
RAF-4TS	12.52	3.39	RAF-8TS	10.57	-2.17
HAT-1TS	43.77	35.46	HAT-6TS	42.26	30.66
HAT-2TS	10.72	2.76	HAT-7TS	14.25	2.18
HAT-3TS	12.45	3.50	HAT-8TS	16.02	2.99
HAT-4TS	8.33	-0.26	HAT-9TS	12.00	-0.14
HAT-5TS	37.83	29.57	HAT-10TS	31.88	19.96
M-P+NO_2	-29.01	-30.62	M-P-S+NO_2	-24.06	-13.86
IM1	-23.82	-33.15	IM1-S	-12.50	-26.39
IM2	4.58	-5.28	IM2-S	14.37	1.06
IM3	-24.09	-33.13	IM3-S	-8.53	-22.25
IM4+H_2O	-3.08	-2.00	IM4-S+HSO_4^-	5.37	5.82
IM5+H_2O	-30.76	-29.78	IM5-S+HSO_4^-	-22.31	-21.96
IM6+H_2O	-21.05	-5.36	IM6-S+HSO_4^-	-12.59	2.47
IM7+H_2O	-21.12	-20.29	IM7-S+HSO_4^-	-12.67	-12.47
IM8+H_2O	-14.90	-14.33	IM8-S+HSO_4^-	-6.45	-6.50

Table S3. The calculated rate constants ($M^{-1}s^{-1}$) between 278 and 318 K in the reaction of MNZ and HO•.

T	k_{RAF-1}	k_{RAF-2}	k_{RAF-3}	k_{RAF-4}	$k_{total-RAF}$	k_{HAT-1}	k_{HAT-2}	k_{HAT-3}	k_{HAT-4}	k_{HAT-5}	$k_{total-HAT}$	k_{total}
278	1.29×10^{10}	3.48×10^{6}	5.35×10^{-7}	9.08×10^{4}	1.29×10^{10}	3.26×10^{c20}	1.01×10^{7}	4.48×10^{5}	4.16×10^{8}	3.57×10^{-15}	4.26×10^{8}	1.33×10^{10}
288	1.01×10^{10}	3.96×10^{6}	1.57×10^{-6}	1.12×10^{5}	1.01×10^{10}	3.04×10^{-19}	1.18×10^{7}	5.41×10^{5}	4.06×10^{8}	2.22×10^{-14}	4.19×10^{8}	1.05×10^{10}
298	8.10×10^{9}	4.49×10^{6}	4.32×10^{-6}	1.36×10^{5}	8.10×10^{9}	2.44×10^{-18}	1.38×10^{7}	6.46×10^{5}	3.98×10^{8}	1.22×10^{-13}	4.13×10^{8}	8.52×10^{9}
308	6.59×10^{9}	5.05×10^{6}	1.11×10^{-5}	1.65×10^{5}	6.59×10^{9}	1.72×10^{-17}	1.59×10^{7}	7.65×10^{5}	3.92×10^{8}	6.02×10^{-13}	4.09×10^{8}	7.00×10^{9}
318	5.44×10^{9}	5.65×10^{6}	2.71×10^{-5}	1.97×10^{5}	5.44×10^{9}	1.08×10^{-16}	1.82×10^{7}	8.97×10^{5}	3.87×10^{8}	2.69×10^{-12}	4.06×10^{8}	5.85×10^{9}

Table S4. The calculated rate constants ($M^{-1}s^{-1}$) between 278 and 318 K in the reaction of MNZ and $SO_4^{\bullet-}$.

T	k_{RAF-5}	k_{RAF-6}	k_{RAF-7}	k_{RAF-8}	$k'_{total-RAF}$	k_{HAT-6}	k_{HAT-7}	k_{HAT-8}	k_{HAT-9}	k_{HAT-10}	$k'_{total-HAT}$	k'_{total}
278	3.76×10^{9}	8.25×10^{6}	1.92×10^{-14}	3.24×10^{6}	3.77×10^{9}	1.39×10^{-18}	2.04×10^{4}	8.20×10^{2}	6.39×10^{5}	2.36×10^{-10}	6.60×10^{5}	3.77×10^{9}
288	2.47×10^{9}	6.86×10^{6}	8.42×10^{-14}	2.96×10^{6}	2.48×10^{9}	9.79×10^{-18}	2.40×10^{4}	9.99×10^{2}	6.61×10^{5}	8.32×10^{-10}	6.86×10^{5}	2.48×10^{9}
298	1.68×10^{9}	5.78×10^{6}	3.35×10^{-13}	2.72×10^{6}	1.69×10^{9}	6.08×10^{-17}	2.80×10^{4}	1.20×10^{3}	6.85×10^{5}	2.70×10^{-9}	7.15×10^{5}	1.69×10^{9}
308	1.17×10^{9}	4.95×10^{6}	1.22×10^{-12}	2.53×10^{6}	1.18×10^{9}	3.36×10^{-16}	3.25×10^{4}	1.44×10^{3}	7.11×10^{5}	8.13×10^{-9}	7.45×10^{5}	1.18×10^{9}
318	8.39×10^{8}	4.28×10^{6}	4.11×10^{-12}	2.36×10^{6}	8.45×10^{8}	1.68×10^{-15}	3.74×10^{4}	1.70×10^{3}	7.38×10^{5}	2.29×10^{-8}	7.77×10^{5}	8.46×10^{8}

Table S5. The calculated half-life ($t_{1/2}$) for MNZ depending on the concentration of •OH of 10^{-9}–10^{-18} mol/L within the temperature range of 278−318 K.

[OH] \ $t_{1/2}$(s) \ T(K)	278K	288K	298K	308K	318K
1.00×10^{-9}	0.052	0.066	0.081	0.099	0.119
1.00×10^{-10}	0.52	0.66	0.81	0.99	1.19
1.00×10^{-11}	5.21	6.57	8.14	9.90	11.85
1.00×10^{-12}	52.10	65.74	813.82	99.01	1185.39
1.00×10^{-13}	5.21×10^{2}	6.57×10^{2}	8.14×10^{3}	9.90×10^{2}	1.19×10^{3}
1.00×10^{-14}	5.21×10^{3}	6.57×10^{3}	8.14×10^{3}	9.90×10^{3}	1.19×10^{4}
1.00×10^{-15}	5.21×10^{4}	6.57×10^{4}	8.14×10^{4}	9.90×10^{4}	1.19×10^{5}
1.00×10^{-16}	5.21×10^{5}	6.57×10^{5}	8.14×10^{5}	9.90×10^{5}	1.19×10^{6}
5.88×10^{-17}	8.86×10^{5}	1.12×10^{6}	1.38×10^{6}	1.68×10^{6}	2.02×10^{6}
3.27×10^{-17}	1.59×10^{6}	2.01×10^{6}	2.49×10^{6}	3.03×10^{6}	3.63×10^{6}
2.15×10^{-17}	2.42×10^{6}	3.06×10^{6}	3.79×10^{6}	4.61×10^{6}	5.51×10^{6}
1.00×10^{-17}	5.21×10^{6}	6.57×10^{6}	8.14×10^{6}	9.90×10^{7}	1.19×10^{7}
1.00×10^{-18}	5.21×10^{7}	6.57×10^{7}	8.14×10^{7}	9.90×10^{7}	1.19×10^{8}

Table S6. The acute and chronic toxicity class (mg L^{-1}).

Classification	Acute toxicity[1]	Chronic toxicity[2]
**Not harmful**	LC_{50}>100 or EC_{50}>100	ChV>10
**Harmful**	10< LC_{50} <100 or 10< EC_{50} <100	1< ChV <10
**Toxic**	1< LC_{50} <10 or 1< EC_{50} <10	0.1< ChV <1
**Very toxic**	LC_{50}<1 or EC_{50}<1	ChV<0.1

[1]Criteria set by the European Union (described in Annex VI of Directive 67/548/EEC);

[2]Criteria set by the Chinese hazard evaluation guidelines for new chemical substances (HJ/T 154–2004).

Table S7. Eoctoxicity values of MNZ and its transformation products to aquatic organisms (mg L^{-1}).

		MNZ	M-P	P1	P2	P3
Log K_{ow}		-0.0028	0.4493	0.6695	0.7191	-0.6873
Acute Toxicity	Fish (LC_{50})	878.33	260.97	1468.34	1836.83	33663.22
	Daphnia (LC_{50})	179.75	79.78	737.35	926.62	14914.02
	Green Algae (EC_{50})	6.92	3.56	330.50	423.28	3982.36
Chronic Toxicity (ChV)	Fish	0.95	0.50	124.16	156.16	2455.54
	Daphnia	3.08	1.37	51.08	65.02	729.11
	Green Algae	2.70	1.45	65.84	85.18	600.16
